# A mixed-methods study to evaluate the feasibility and preliminary efficacy of delivering the optimal health program (OHP) for youth at clinical high risk (CHR) for psychosis: A study protocol

**DOI:** 10.1371/journal.pone.0306968

**Published:** 2024-07-18

**Authors:** Muhammad. Omair Husain, Lisa D. Hawke, Yun Lu, Nicole Kozloff, Gillian Strudwick, Michael Kiang, Wei Wang, David Castle, George Foussias

**Affiliations:** 1 Department of Psychiatry, University of Toronto, Toronto, Ontario, Canada; 2 Centre for Addiction and Mental Health, Toronto, Ontario, Canada; 3 Institute of Health Policy, Management and Evaluation, University of Toronto, Toronto, Ontario, Canada; 4 Department of Psychiatry, University of Tasmania, Tasmania, Australia; 5 Centre for Mental Health Service Innovation, Statewide Mental Health Service, Tasmania, Australia; The AIDS Support Organization, UGANDA

## Abstract

Individuals with clinical high risk (CHR) for psychosis experience significant distress, impaired general functioning and a high lifetime risk of self-harm and attempted suicide. The CHR period is an important phase in an individual’s mental health where appropriate interventions may reduce the risk of progression to several negative outcomes, including the development of schizophrenia. Given that up to 80% of individuals with CHR have another diagnosable mental illness and almost half experience poor psychosocial functioning, developing interventions that address psychosocial functioning in young people with CHR is of great importance. This mixed-methods study aims to employ qualitative and quantitative methods to adapt an evidence-based comprehensive psychosocial and mental health self-efficacy program, the Optimal Health Program (OHP), and evaluate the feasibility, acceptability and preliminary clinical efficacy in young people with CHR. We aim to recruit 30 CHR participants (age 16–29 years) in a single-arm 12-week exploratory clinical trial. Feasibility metrics will include recruitment, retention, and data completion rates. Acceptability will be informed by the Client Satisfaction Questionnaire. Clinical assessments (psychosis spectrum symptoms, depression, and anxiety), functional measures, and cognitive outcomes will be completed at study entry and repeated post-intervention at 12-weeks. We will run pre-post test data analysis to examine changes following engagement in the OHP intervention. Qualitative interviews will be conducted post-intervention to further evaluate the acceptability of the intervention and the trial design, and will be analyzed using thematic analysis. OHP may enhance the long-term mental health, well-being and functioning of CHR youth. However, the intervention must first be adapted to a CHR population; then, the feasibility and preliminary efficacy of delivering an intervention tailored around the varied needs of the CHR group must be established before a larger-scale appropriately powered study is pursued.

**Trial registration:** The trial is registered with ClinicalTrials.gov NCT05757128.

## Introduction

Categorization of the clinical high risk (CHR) for psychosis was introduced to inform the early detection of individuals at elevated risk of developing psychosis, to provide indicated interventions and prevent progression to more severe outcomes [1]. Around a third of CHR individuals develop a psychotic illness in the ensuing three years, and many of those who do not develop a psychotic illness per se, continue to experience attenuated psychotic symptoms, poor functioning as well as other psychiatric comorbidities [2]. Young people with CHR experience significant distress and have a high lifetime risk of self-harm and attempted suicide, estimated at 49% and 18% respectively [3]. The CHR period is a critical phase where interventions may reduce the risk of progression to several negative outcomes, including schizophrenia. Given the high prevalence of anxiety, depression and substance use disorders in CHR, treatment strategies need to be adapted to address these issues [4]. Adopting a wider concept of therapeutic treatment to prioritize social recovery and functioning is a logical step towards prioritizing patient-centered goals [5, 6] and is consistent with trans-diagnostic approaches to emerging mental illness [7]. Depression and anxiety co-morbidity in CHR are linked to increased suicidality, self-harm, and poorer functioning, necessitating prompt assessment and treatment [8]. In young people (aged 15–29 years) suicide is the second leading cause of death worldwide [9] and psychosis-like symptoms are associated not only with comorbid mental health problems and poor functioning, but also with suicide [10]. Shifting focus to improving functional outcomes and enabling CHR youth to access appropriate transdiagnostic interventions has a potentially profound public health impact.

Most published CHR intervention trials have focused on transition to psychosis, psychotic symptoms, and associated distress, with little emphasis on functional outcomes across diagnoses [11, 12]. Nonetheless, meta-analyses support interventions delivered during CHR in effectively delaying transition to a full-blown psychotic illness [13] and reducing subthreshold symptoms at 12 months [14]. While these findings are important, studies of discrete interventions do not reflect real-world complexity where multifaceted treatments are adapted to individual needs [13]. Broader multicomponent interventions are required for individuals with CHR, encompassing biological and psychosocial aspects as well as personalizing them to the needs of the individual in their sociocultural and developmental context. In contrast to later-stage illnesses, in the CHR state there is uncertainty about illness trajectory, and this favors a ‘light touch’ approach and transitioning to individualized, multicomponent treatments where required, responsive to the heterogeneity seen in this population. In large cohorts of people with CHR, approximately a third remit spontaneously and require no further treatment [15] but more commonly people develop mood disorders, anxiety and substance use disorders [2, 7]. There is strong evidence from a large, prospectively obtained, help-seeking cohort that almost three quarters of CHR have another clinical diagnosis of mental illness that warrants treatment [4]. This is consistent with a recent systematic review that found up to 80% of CHR patients had another diagnosable mental illness, and almost half experienced poor psychosocial functioning up to six years after first seeking help [16]. For these reasons, developing and testing multi-component interventions that address complex intersecting needs in developmentally informed ways, including youth and family-engaged intervention development and evaluation projects, reflecting the dynamic nature of youth development are needed for young people with CHR.

This protocol details the adaptation and delivery of a comprehensive, evidence-based, ready-to-go psychosocial and mental health support program, the Optimal Health Program (OHP), to enhance mental health and support functional recovery for individuals with CHR. The OHP is a self-efficacy program, delivered virtually or in-person, which provides a framework to address the psychosocial and mental health of individuals, adopting a patient-centered approach that empowers the individual to be at the center of their own health management [17]. Developed specifically to support people with mental health issues, it also encompasses physical health and substance use management strategies. The OHP comprises psychosocial supports and development of a skill-base designed to build resiliency and maintain optimal health [17]. The efficacy of OHP has been shown in improving depression, anxiety, and quality of life in community mental health settings, as well as in individuals with chronic physical illnesses [18].

Failure to provide adequate and timely mental health support to CHR individuals is a missed opportunity to avert progression to more severe outcomes, at which stage affected individuals are more likely to engage with acute mental health services. Given the high prevalence of poor functioning within the CHR population and the pivotal developmental stages CHR presents, effective treatment is likely to deliver substantial economic and social gains, reducing disability and enhancing productivity. OHP co-developed by youth for youth, may improve long-term functional outcomes, including vocational and educational outcomes, and enhance quality of life, while reducing reliance on institutionalized care. Thus, we will adapt and test a version of OHP for CHR (OHP-CHR) in terms of feasibility and preliminary efficacy.

### Aims and objective

The primary objective of the study is to adapt an existing comprehensive resilience-building psychosocial and mental health support program, the Optimal Health Program (OHP), for CHR youth and collect feasibility evidence for a trial testing its ability to improve functioning, reduce distress, and increase resilience in youth with CHR. Specific aims of the study include 1) to adapt an existing, effective, validated psychological intervention for use in CHR youth; 2) to evaluate the acceptability of OHP-CHR and the feasibility of conducting a clinical trial of OHP in CHR youth; 3) to assess the preliminary efficacy of OHP-CHR in enhancing resilience, reducing depression and anxiety, and improving functioning in CHR youth, employing a single-arm exploratory clinical trial design.

## Methods

### Study design and settings

**Fig 1:**
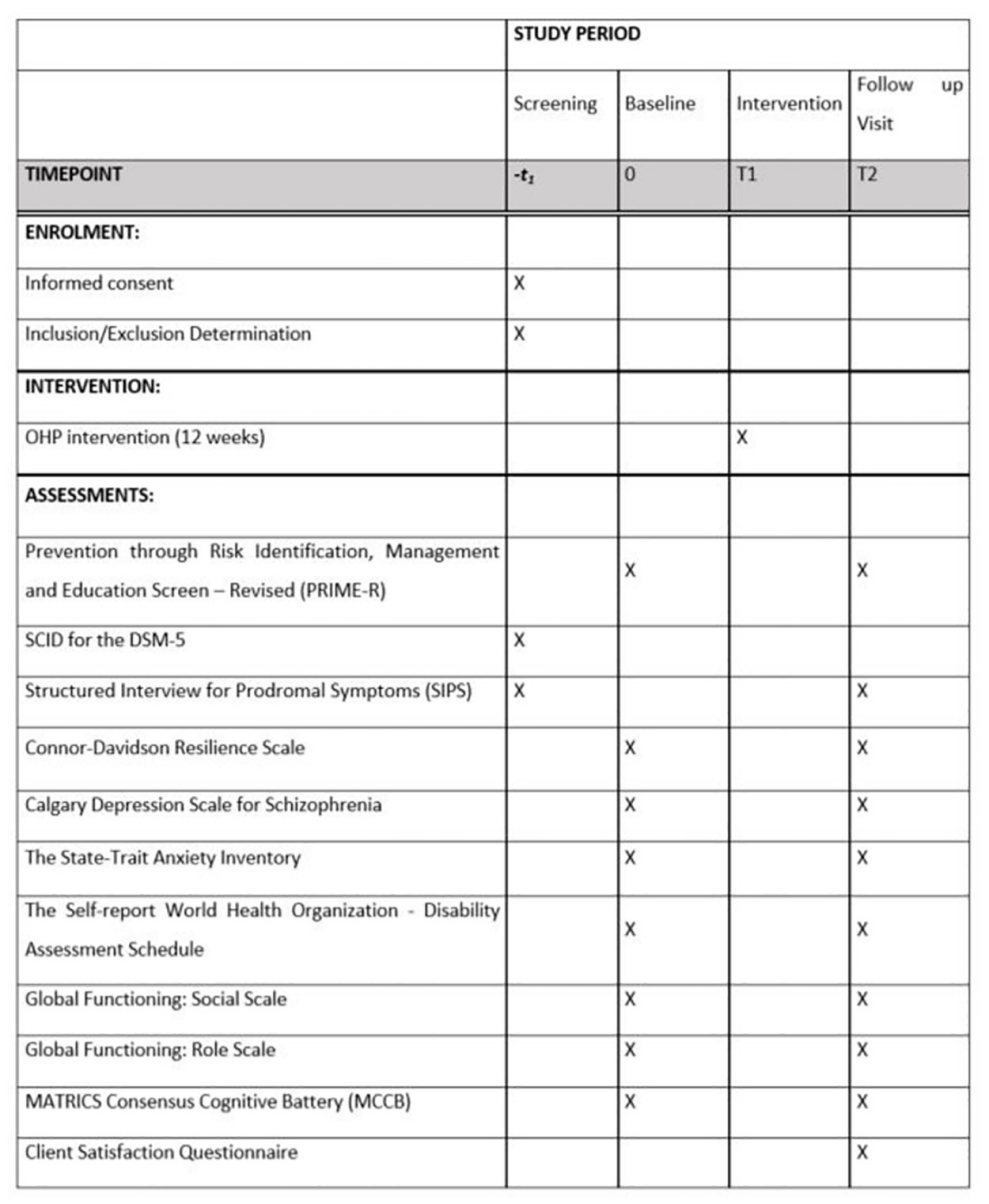
SPIRIT schedule of enrollment, intervention, and assessments.

This mixed-methods study employs qualitative and quantitative methods to adapt the OHP intervention and test the feasibility and acceptability of OHP in individuals with CHR. The design of the study has been informed by the Medical Research Council (MRC) framework for developing and evaluating complex interventions [19]. The MRC framework guides researchers in the design, methodology and conduct of research relating to the development of complex interventions. We will conduct a single-arm, pre/post intervention study, where OHP-CHR will be delivered over 12-weeks. We plan to enroll 30 participants over two years. Outcome measures will be completed at study entry and immediately post-treatment. For the qualitative component of the study, we will conduct 15 post-intervention interviews. The Research Ethics Board (REB) at the Centre for Addiction and Mental Health has provided ethics approval for this study (REB number: 063/2022). The SPIRIT schedule of enrollment, intervention, and assessments is included as Table 1. A graphic timeline is provided in Fig 1. The trial is registered with ClinicalTrials.gov (NCT05757128). The SPIRIT checklist is included in S1 Checklist. A full accounting of evaluation and unabridged protocol approved by the REB is available in S1 Protocol (REB number: 063/2022, April 5^th^ 2023, Version 2.0). Any changes to the trial protocol, including any modifications of the study objectives, design, population, sample sizes, or procedures, will be approved by the REB prior to implementation. Research personnel will explain all study procedures to all participants and written informed consent will be obtained prior to their enrolment in the study.

**Table 1:** SPIRIT schedule of enrolment, interventions, and assessment.

	STUDY PERIOD			
	Screening	Baseline	Intervention	Follow up Visit
TIMEPOINT	*-t*_*1*_	0	T1	T2
**ENROLMENT:**				
Informed consent	X			
Inclusion/Exclusion Determination	X			
**INTERVENTION:**				
OHP intervention (12 weeks)			X	
**ASSESSMENTS:**				
Prevention through Risk Identification, Management and Education Screen–Revised (PRIME-R)		X		X
SCID for the DSM-5	X			
Structured Interview for Prodromal Symptoms (SIPS)	X			X
Connor-Davidson Resilience Scale		X		X
Calgary Depression Scale for Schizophrenia		X		X
The State-Trait Anxiety Inventory		X		X
The Self-report World Health Organization—Disability Assessment Schedule		X		X
Global Functioning: Social Scale		X		X
Global Functioning: Role Scale		X		X
MATRICS Consensus Cognitive Battery (MCCB)		X		X
Client Satisfaction Questionnaire				X

The study will include (1) Adaptation of OHP for CHR youth, co-produced with a lived-experience youth engagement group; (2) Screening phase for eligibility and the informed consent process; (3) baseline assessments; (4) 12-week OHP-CHR intervention; (5) post-treatment outcome assessments and at 12 weeks (Time point T2); (6) post-intervention semi-structured qualitative interviews at 12 weeks (T2). Study participants will be from the Centre for Addiction and Mental Health (CAMH), Canada, and the clinical trial will also be conducted there. The tentative start and end date of the recruitment period for this study is from 24/08/2023 to 09/14/2024.

### Participants: Recruitment and eligibility

CHR youth will be recruited with a focus on diversity across genders, ethnic origins, age, and other sociodemographic characteristics. Study participants will be recruited from the Slaight Family Centre for Youth in Transition (SFCYT) at CAMH via two mechanisms: 1) the Slaight centralized clinical research recruitment strategy in SFCYT where a core team of clinical research staff identify and engage all patients receiving early psychosis services in clinical research, providing streamlined triaging of patients to appropriate studies; 2) the CAMH-wide Clinical Engagement and Research Recruitment (CLEARR) infrastructure that enables early identification and engagement in research for all new patients presenting for care at CAMH.

#### Inclusion criteria

The participant must meet all of the following inclusion criteria to be eligible for this study: (1) be 16–29 years old; (2) be competent and willing to consent to study participation; and (3) meets CHR criteria for a psychosis risk syndrome based on the Structured Interview for Psychosis Risk Syndromes (SIPS) [20] either currently or at some point in the past 3 years.

#### Exclusion criteria

An individual who meets any of the following criteria will be excluded from participation in this study: (1) diagnostic and Statistical Manual of Mental Disorders (DSM-5) diagnosis of psychotic disorder (e.g., schizophrenia spectrum disorder, mood disorder with psychotic features); (2) diagnosis of intellectual disability previously documented in the patient chart; (3) severe developmental disorder; (4) acute suicidality requiring immediate intervention.

### Procedure

**Fig 2:**
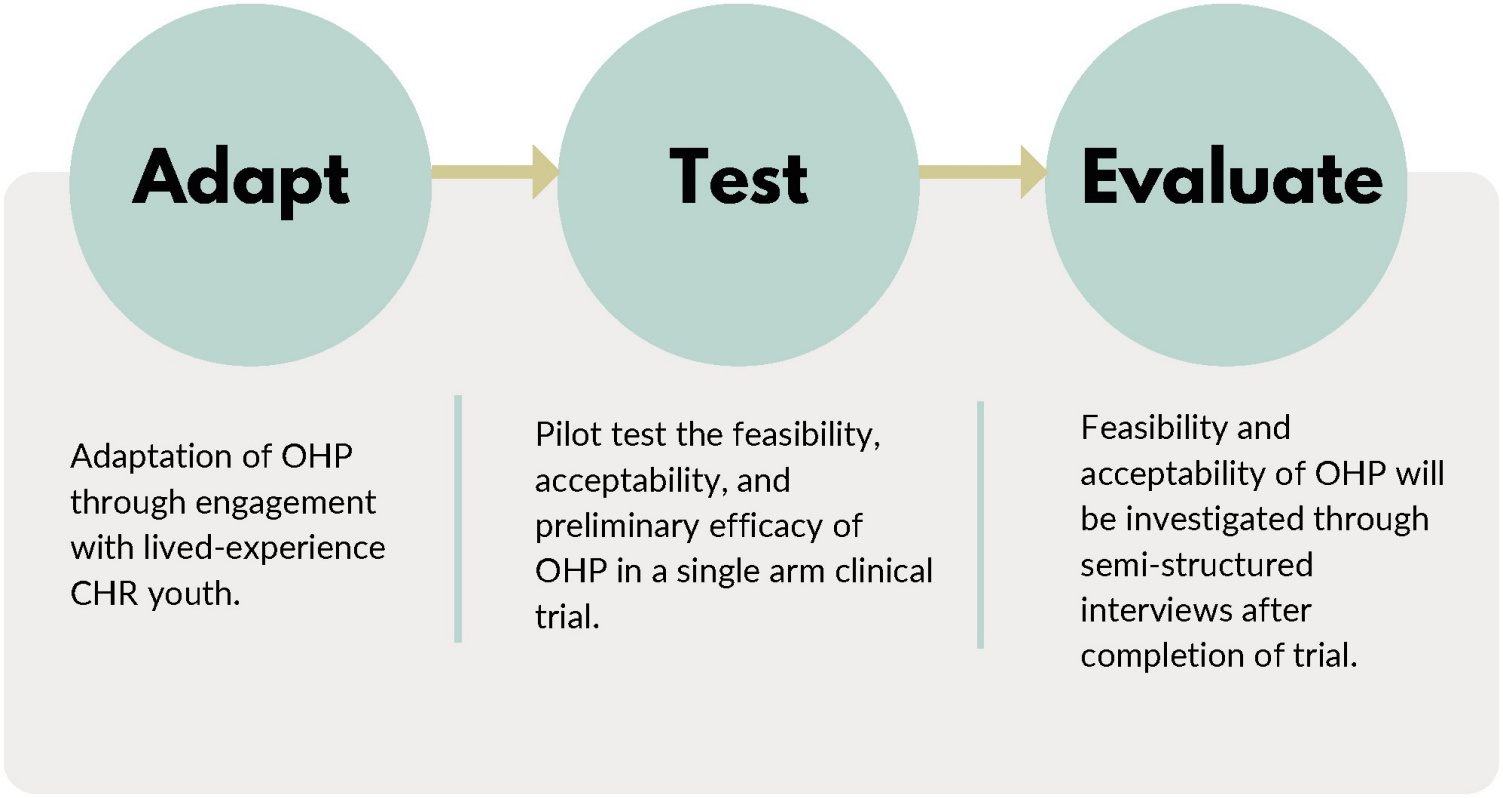
Three consecutive phases of the study design.

The present study consists of three consecutive phases (See Fig 2).

#### Phase 1: Adaptation of OHP through engagement with CHR youth

This phase will consist of the adaptation of OHP for Canadian CHR youth through engagement with youth with lived experience. Lived-experience youth will advise on iterative adaptations of OHP through 8 feedback sessions. Researchers will present the OHP model, walk through the content of each session to gain their feedback on youth- and diagnostic-relevant adaptations, explore attitudes and beliefs about the support needs of CHR youth, impact of illness and priorities/preferences for delivery including session duration, location, practitioner preference and role of the caregiver to inform developmentally appropriate interventions. Youth will be engaged in all stages of the pilot trial in the form of a youth advisory group and will also help in knowledge translation of study results. Patient Engagement in Research conceptual framework will be followed to guide engagement and youth will be compensated for their time [21].

#### Phase 2: Pilot feasibility testing of OHP

OHP-CHR will be pilot tested for feasibility, acceptability, and preliminary efficacy in a single arm pre-post intervention trial. Following provision of written informed consent, participants will be assessed for suitability for inclusion in the study based on the inclusion and exclusion criteria. The clinical, functional, and neuropsychological assessments will be performed at baseline once participants are enrolled in the study. These assessments will be scheduled on the same day or separate days depending on participant preference. Participants will then receive 12 weeks of OHP intervention. The assessments will be repeated at the end of the 12-week intervention.

#### Phase 3: Qualitative evaluation of OHP

Feasibility and acceptability of OHP-CHR will be assessed through semi-structured interviews with the participants after completion of the trial. Semi-structured interviews will be conducted to obtain views of OHP-CHR, treatment target preferences, and experiences of assessment schedules. Interview data will also be collected on experiences participating remotely/technical experience, overall perception of the program, and barriers/facilitators to participation. An interview guide will be developed from existing literature, together with feedback from the youth advisory group, and will consist of key questions with optional prompts.

### Intervention

**Fig 3:**
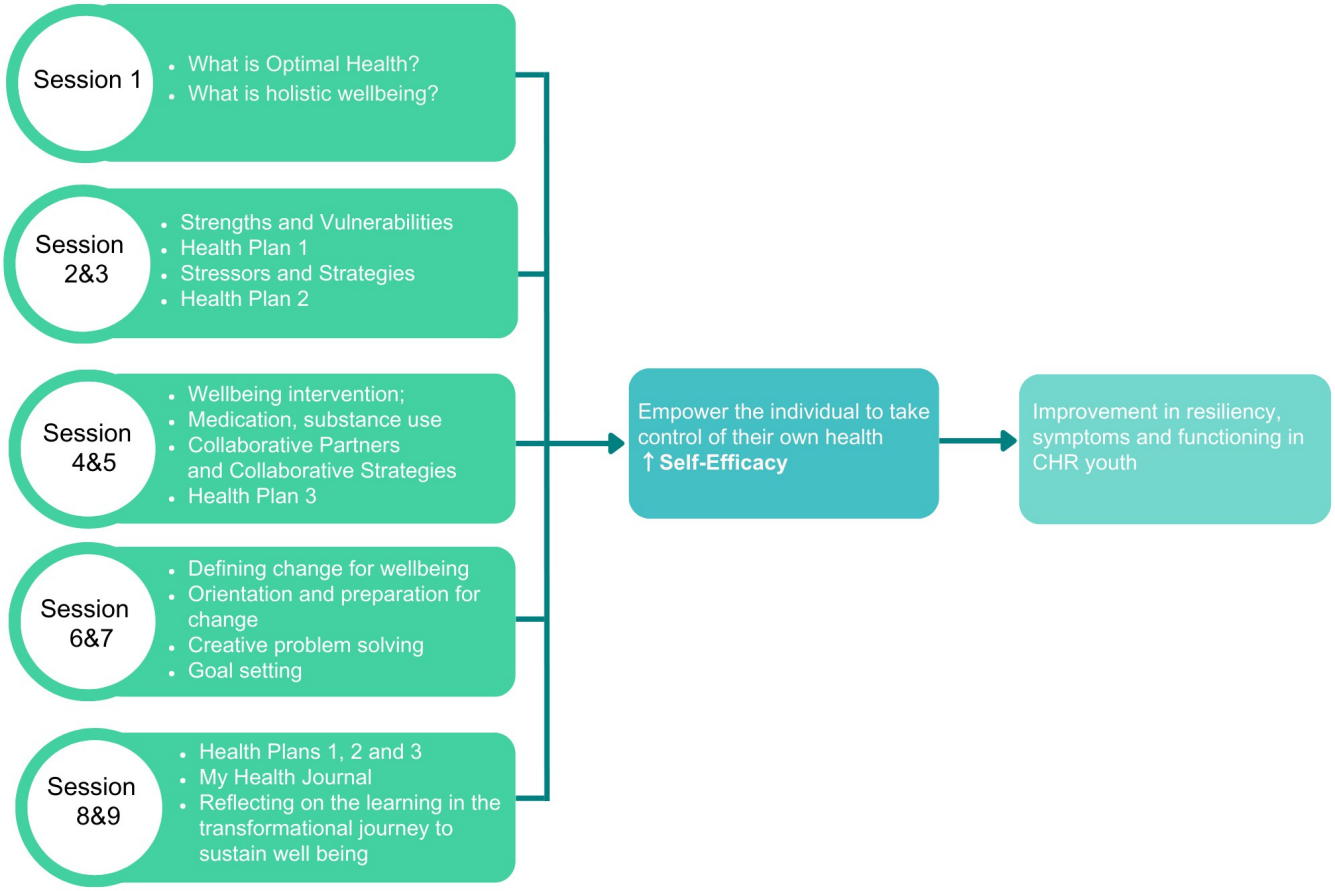
Structure of OHP intervention and pathway to outcome.

The OHP intervention is a psychosocial management program that will be adapted to the CHR population and will be accompanied by a structured workbook (in paper-based and PDF format). The OHP is a program delivered over 9 sessions (Fig 3) weekly for the first 6 weeks and every two weeks for the remaining 6 weeks. The sessions will be delivered one on one (either face-face or virtually based on participant preference), and each session will last approximately 1 hour in duration. A facilitator will deliver each session. All facilitators will complete a 2-day training workshop. Following the workshop, regular supervision and fidelity checks will be provided. Participants may receive routine mental healthcare including follow ups by a psychiatrist or case management. Participants will be withdrawn from the study should they develop or are found to have any condition that might compromise safety. Other reasons for withdrawing individuals from the study may include any of the following: major protocol violation; participant loss to follow-up; withdrawal of consent; any participant may be discontinued from the study at the discretion of the investigators if it is deemed to be in the best interest of the participant.

The OHP has three components: 1) assessment and engagement; 2) therapy sessions, and 3) maintenance integration.

1. **Assessment/engagement**. The assessment aims to screen for issues that may act as barriers to treatment and to develop a pathway that maximizes the individual’s engagement in the program.
2. **Therapy sessions**. The cornerstone of the OHP intervention is the one-on-one based work which runs over the intervention period, followed by a relapse prevention component (final session). Sessions are based on a novel version of the stress vulnerability model (stress vulnerability/self-efficacy), utilizing individual self-efficacy and self-reliance as part of the process. OHP provides core components of therapeutic interventions that have established efficacy across a wide range of mental health diagnoses. These consist of psychoeducation, coping and relapse prevention strategies, and other skills that can be usefully employed to promote positive wellbeing and ongoing mental health maintenance. OHP has a specific module focusing on alcohol and other drugs; stages of change, impact of substance use, implementation of harm minimization strategies, engaging with support groups, change enhancement and problem-solving.
3. **Maintenance integration**. Via an individualized OHP journal, participants can chart stressors, early warning signs, coping strategies, supports and other factors that influence the course and maintenance of their health. It places them at the center of their treatment and provides them with effective skills to maintain wellbeing, manage psychological distress and the ability to facilitate good communication between themselves and others involved in the maintenance of their mental health (e.g., clinicians, family physicians, mental health workers). The journal thus: a) allows the participant to identify the supports and services they can draw upon; b) facilitates on-going skills development and provides empowerment and ownership of health and wellbeing; and c) allows monitoring of mental health and physical health parameters over time.

### Safety and adverse events

There are no anticipated risks to the participants as the intervention is a ‘light-touch’ psychosocial intervention delivered in addition to usual care. Anyone identified with acute psychosis or at acute risk of suicide will be assisted to immediately contact local mental health professionals. If required, the research team will accompany the participant to the local mental health service provider. All assessments will be completed in accessible, private, and appropriate venues, either in person or remotely that suit the needs and preferences of participants. The appointments will be scheduled at times convenient to participants, considering education, household commitments and employment commitments. Participants will not be exposed to a risk of physical and mental harm that is greater than that typically encountered in normal life and the recruitment materials will direct participants to relevant supports if participation raises any concerns.

### Outcome measures

#### Primary outcomes

Feasibility and acceptability are the primary outcomes of this study. These outcomes have been informed by the MRC framework for developing and evaluating complex interventions [19]. Feasibility outcomes will include recruitment, adherence, retention, attrition, and completeness of outcome schedule at each time point. Acceptability will be informed by the Client Satisfaction Questionnaire (CSQ-8) [22]. Semi-structured qualitative interviews conducted at the end of the intervention will also provide data on feasibility and acceptability.

#### Secondary outcomes

Several secondary outcomes will be conducted on clinical, functional, and neuropsychological assessments. These will be done at baseline and will be repeated at approximately 12-weeks (up to 18 weeks after baseline).

*Clinical outcomes*. Psychiatric diagnoses will be confirmed using the Structured Clinical Interview (SCID) for DSM-5 at baseline. Psychosis symptoms will be assessed using PRIME-Revised [23], Structured Interview for Prodromal Symptoms (SIPS) [20]. Calgary Depression Scale for Schizophrenia (CDSS) [24] and the State-Trait Anxiety Inventory (STAI) [25] will be used to assess mood symptoms and anxiety symptoms respectively.

*Functional outcomes*. For functional outcomes, the Self-report World Health Organization—Disability Assessment Schedule (WHO-DAS 2.0, self and proxy versions)—a 12-item generic assessment instrument for health and disability [26] and Global Functioning (GF): Social and Role Scales [27] will be used.

*Cognitive outcomes*. Seven cognitive domains: (1) Speed of Processing; (2) Attention/Vigilance; (3) Working Memory; (4) Verbal Learning; (5) Visual Learning; (6) Reasoning and Problem Solving; and (7) Social Cognition will be assessed using MATRICS Consensus Cognitive Battery (MCCB) [28]. Resilience will be assessed through Connor-Davidson Resilience Scale (CD-RISC) [29].

## Data collection and data management

The principal investigator (PI) will meet weekly with study personnel to review accrued data, data confidentiality, and adherence to protocol design, recruitment, and participant complaints. During meetings, the study PI will also review the enrollment data, the accrual and integrity of clinical, and neurocognitive data, and any adverse event associated with the various components of the study. Based on these reports, we will determine if there has been any change in the benefit-to-risk ratio of the clinical and cognitive assessment components of the study. Participant Study File (PSF) will be completed for each participant enrolled in the study. All information recorded on the PSFs for this study will be considered the participant’s source documentation. Source data from the PSF will be collected and documented on paper and/or using the “Electronic Data Capture” solution (REDCap) [30] on a secure CAMH server. A participant screening and enrollment log, noting reasons for screen failure, where applicable, will be maintained for all participants.

Study data will be entered in a secure database (REDCap). At point-of-entry, data values will undergo consistency edits (e.g., ID validation, range verification, duplicate detection) and personnel will correct any errors. Data management staff will run logic error programs to check for accuracy and irregularities within and across data structures. Quality assurance checks will be conducted daily by the personnel, as well as biweekly by data management staff. To reduce the incidence of missing data during data acquisition, we will use several strategies available in REDCap, including marking data fields as required and use of the Data Quality module to run regular queries for ALL missing data. This will alert study personnel/PI of missing values and regular data audits using REDCap’s Data Resolution Workflow, allowing a data auditor to open queries (based on the Data Quality Module) for data entry personnel to respond to (and leaving an audit trail that can be tracked). For the relevant assessments, the study REDCap database will also retain form statuses to reflect whether an assessment was collected in-person or virtually.

The hard copies of data will be stored in a locked filing cabinet in a locked office to further protect participant anonymity. Data auditing, entry, and quality control will be carried out routinely. Regularly scheduled communication between the study team and the PI will clarify any inconsistencies and ambiguities in the data. Additional communication will be conducted as needed. All data pertaining to a participant’s involvement in this study will be coded and stored securely (in locked offices or secure database/server). In unusual cases, a participant’s research records may be released in response to a court order. If the research team learns that a participant or someone with whom the participant is involved with is in danger or harm, an investigator will inform the appropriate agencies as per legal or regulatory requirements. Throughout the study, data and all appropriate documentation will be maintained according to current regulations and stored for a minimum of 15 years after the completion of the study.

A schedule of enrolment, intervention, and assessment is detailed in Table 1.

### Participant confidentiality

All personal study participant data collected and processed for the purposes of this study will be managed by the investigators and their staff. Adequate precautions to ensure the confidentiality of those data, and in accordance with applicable national and local laws and regulations on personal data protection will be used (in accordance with CAMH policies, PHIPA, Tri-Council Policy Statement (TCPS2) and the International Conference on Harmonization Guideline for Good Clinical Practice (ICH GCP) requirements). There is a potential risk of breach of confidentiality that is inherent in all research protocols. Breach of confidentiality will be minimized by the staff who will maintain research data (identified only by participant code number not personal identifying information) in separate charts and a dedicated password protected electronic database. A list of participant names, their ID numbers, and information about how they can be reached will be kept in a separate locked cabinet with access only to study personnel authorized by the PI. Procedures have been established, and will be followed, to minimize the risk of breach of confidentiality. Procedures to maintain confidentially include: (1) formal training sessions for all research personnel emphasizing the importance of confidentiality; (2) specific procedures developed to protect participants’ confidentiality, and (3) formal mechanisms limiting access to information that can link data to individual participants. All information obtained from participants will be kept as confidential as possible. Computer based files/data will be entered into password-secured databases and paper-based files will be stored in a secure location. Data storage and management will abide by confidentiality regulations of the REB. The ethics committee granting approval to this study will be granted direct access to the study participants’ original medical records for verification of study procedures and/or data, without violating the confidentiality of the participants, to the extent permitted by the law and regulations. Participants will not be identified by name in any publication or presentations at meetings of research results. Results will be published as group data without the use of characteristics that would identify individual participants.

### Sample size and statistical analyses

#### Sample size

We aim to recruit 30 participants and estimate to account for 20% loss to follow up [25, 31]. The sample size was informed by the published literature, which suggests 24–50 participants for feasibility studies [20, 23]. The proposed sample size will provide reasonably reliable quantitative estimates for the targeted feasibility measures. The margins of error are ±12.7% for recruitment rate, ±14.3% for adherence/retention/attrition, and ±12.0% for completeness of outcomes. The minimum detectable effect size for a pre-post test is 0.60 (Cohen’s d, power of 80% and a 5% two-tailed significance level). Participants from the single-arm study will be invited for the qualitative evaluation. The sample size for the qualitative evaluation will be determined by the principle of saturation [32]; we estimate that 15 post-intervention interviews will need to be conducted [33].

#### Statistical methods

*Missing data*, *data preparation*, *and monitoring*. We will implement strategies including data check to minimize missing data. A data monitoring committee will not be required for the current proposed trial because it does not have any of the following features: major safety concern, unknown risks, long-term follow-up, or double-blind treatment assignment.

*Quantitative analysis*. Analyses for the primary outcome of feasibility will be reported as descriptive statistics including recruitment rate, participant retention, attendance rates to interventions sessions, and completeness of outcome measures. For acceptability, CSQ-8 group mean scores and standard deviations will be reported. Distributional characteristics of the outcome measures will be assessed for ceiling and floor effects and rates and patterns of missing values. To address the aim of exploring the preliminary efficacy of OHP-CHR, we will run a pre-post repeated-measures analysis of covariance to assess for any changes in resilience (CD-RISC score), depression and anxiety symptoms (CDSS and STAI score), cognition (MATRICS score) and functioning (WHO-DAS 2.0 and GF: Social and Role Score) following intervention. Findings will be reported with both p values and 95% confidence intervals and all analysis will be considered exploratory. Given the small sample size, appropriate to a feasibility trial, we will also calculate effect sizes (Cohen’s d), which we will compare with the effect sizes from previous OHP trials to gain a sense of the effect that might be achieved in the full-scale trial.

*Qualitative analysis*. The interviews with the participants after completing the study will explore the acceptability of the intervention. All interview data will be transcribed verbatim. Data will be analyzed using the five stages of framework analysis [33]. Rigor will be ensured by maintaining an audit trail to foster dependability of the process. Confirmability and credibility will be ensured through the involvement of two independent coders in the process of data analysis and discussion of emerging themes. Consensus of the final theoretical framework, reflexivity, and the impact of the researchers on recruitment, data collection and analysis will also be considered [34]. Qualitative results will be interpreted in the context of the quantitative findings.

## Discussion

Young people with CHR experience significant distress, have impaired functioning and an elevated lifetime risk of self-harm and attempted suicide. The prevention of mental health problems in young people is a crucial priority for the reduction of personal and societal burden attributed to mental illness globally. Therefore, there is a clear need for novel and efficacious treatment strategies. This study will engage youth in the development of an intervention that has the potential to address the multiple comorbidities and overlapping symptom clusters in young people with CHR. Their strategy will address the requirement for treatment to meet the evolving needs of people at CHR, addressing pleomorphic and fluid symptoms sets which improve clinical outcomes and functioning in this group.

To our knowledge there are no resilience-building health and mental health programs co-developed by youth with lived experience that are clinically adapted to meet the needs of CHR youth. This study has the potential to inform the implementation of evidence-based interventions in Canada and elsewhere. Few specialized CHR programs exist in Canada, and a virtually delivered intervention has the potential to reach many individuals with CHR across the country who would otherwise not have access to care. Virtual delivery of OHP also offers a discreet and socially acceptable way of accessing care, even for youth who may be hesitant to seek mental health support. OHP has been associated with improved health, functioning and reduced health care costs in adult mental health service users and could potentially greatly impact the long-term mental health, well-being and functioning of CHR youth.

A strength of this study is the involvement of youth with lived-experience of psychosis-risk in the development and delivery of this clinical trial. This approach will help to ensure that the OHP CHR intervention is relevant and tailored to specific needs and priorities of CHR youth. Additionally, the virtual delivery of the intervention increases accessibility, especially for those in remote areas and individuals hesitant to seek traditional mental health support. However, the study has limitations. The single-arm study design with lack of a control group, makes it challenging to attribute observed changes directly to the experimental intervention. The primary focus of this study is the adaptation and evaluation of feasibility/acceptability of the OHP-CHR intervention rather than the evaluation of clinical efficacy. A larger confirmatory clinical trial employing a randomized controlled design will be needed in the future to evaluate clinical efficacy. Before this, the intervention must first be adapted to the CHR population; then, the feasibility and preliminary efficacy of delivering an intervention tailored around the varied needs of the CHR group must be established before a larger-scale appropriately powered study is pursued.

## Supporting information

S1 Checklist SPIRIT 2013 checklist. (DOC)

S1 Protocol (DOCX)

S1 File (DOCX)
