# Supplementary material for: A mixed-methods study to evaluate the feasibility and preliminary efficacy of delivering the optimal health program (OHP) for youth at clinical high risk (CHR) for psychosis: A study protocol
Source: PLoS One. 2024 Jul 18;19(7):e0306968. doi: 10.1371/journal.pone.0306968 (PMC11257342; doi:10.1371/journal.pone.0306968)
Supplement: S1 Protocol — (DOCX) [file pone.0306968.s002.docx]

**Optimizing mental health for young people at Clinical High Risk for psychosis (CHR)**

**Protocol Identifying Number (if applicable): *<Number>***

**Principal Investigator: Dr. Omair Husain**

**Funder: Miner’s Lamp Innovation Fund**

**Version Number: *2.0***

**Version Date: *5-APR-2023***

**Table of Contents**

STATEMENT OF COMPLIANCE 3

INTRODUCTION 4

1.1 Background 4

1.2 Study Intervention 5

1.3 Clinical Data to Date 6

1.4 Risks/Benefits 7

1.0 Study Objectives 7

1.1 Specific Aim 1 8

1.2 Specific Aim 2 8

1.3 Specific Aim 3 8

3.0 STUDY DESIGN 8

3.1 Overall Design 8

3.2 Patient Engagement 9

3.3 Quantitative methods 9

3.4 Qualitative methods 9

3.5 Outcome Assessments 10

4.0 PARTICIPANT SELECTION AND WITHDRAWAL 10

4.1 Target Population 10

4.2 Participant Recruitment and Screening 10

4.3 Eligibility Criteria 12

4.3.1 Inclusion Criteria 12

4.3.2 Exclusion Criteria 12

4.4 Participant Withdrawal Criteria 12

6.0 RESEARCH PROCEDURES 13

6.1 Research Visits 13

6.2 Schedule of Events 15

7.0 STATISTICAL PLAN 16

7.1 Sample Size Determination 16

7.2 Statistical Methods 16

8.0 SAFETY AND ADVERSE EVENTS 16

9.0 DATA MANAGEMENT, RETENTION AND INTEGRITY 17

10.0 ETHICAL CONSIDERATIONS 18

10.1 Research Ethics Board (REB) Approval 18

10.2 Informed Consent Process & Documentation 18

11.0 PRIVACY AND CONFIDENTIALITY 19

12.0 FUNDING 20

13.0 REFERENCES 20

# STATEMENT OF COMPLIANCE

This study will be carried out in accordance with the following:

- International Conference on Harmonisation Good Clinical Practice (ICH GCP)
- Tri-Council Policy Statement 2018 (TCPS 2)
- Personal Health Information Protection Act (PHIPA), 2004; Chapter 3 Schedule A (PHIPA) and applicable regulations
- Institutional and REB policies and procedures

________________________________ _____________________________

Signature of PI Date

# INTRODUCTION

## Background

Categorization of the clinical high risk (CHR) for psychosis was introduced to inform the early detection of individuals at higher risk of developing schizophrenia, to provide indicated interventions and prevent progression to more severe outcomes ^[1]^. Even those who do not develop psychosis continue to experience attenuated psychotic symptoms, poor functioning as well as other psychiatric comorbidities ^[2]^. Young people with CHR experience significant distress and have a high lifetime risk of self-harm and attempted suicide, estimated at 49% and 18% respectively ^[3]^. The CHR period is a critical juncture where interventions may reduce the risk of progression to several negative outcomes, including schizophrenia. Given the high prevalence of anxiety, depression and substance use disorders in CHR, treatment strategies need to be refocused on addressing these issues ^[4]^. Adopting a wider concept of therapeutic treatment to prioritize social recovery and functioning is a logical step towards prioritizing patient-centered goals ^[5, 6]^ and is consistent with trans-diagnostic approaches to emerging mental illness ^[7]^. Depression and anxiety co-morbidity in CHR are linked to increased suicidality, self-harm and poorer functioning, necessitating prompt assessment and treatment ^[7]^. In young people (aged 15-29 years) suicide is the second leading cause of death worldwide ^[8]^ and psychosis-like symptoms are associated not only with comorbid mental health problems and poor functioning but also with suicide ^[9]^. Shifting focus to improving functional outcomes and enabling CHR youth to access appropriate transdiagnostic interventions has a potentially profound public health impact.

Conventional CHR intervention trials, however, have focused on transition to psychosis, psychotic symptoms, and associated distress with little emphasis on functional outcomes across diagnoses ^[10, 11]^. Nonetheless, meta-analyses support interventions delivered during CHR in effectively delaying transition to a full-blown psychotic illness ^[12] [10]^, reducing psychotic-like symptoms at 12-months ^[10]^ and improving functional outcomes. While these findings are important, studies of discrete interventions do not reflect real-world complexity where multifaceted treatments that are adapted to individual needs are indicated ^[13]^. We would argue that broader multicomponent interventions are required for individuals with CHR, encompassing biological and psychosocial aspects as well as personalizing them to the needs of the individual in their sociocultural and developmental context. In contrast to later-stage illnesses, during CHR there is uncertainty about illness trajectory that favors a ‘light touch’ approach and transitioning to individualized, multicomponent treatments is more consistent with heterogeneity seen in this population. In large cohorts of people with CHR, approximately a third will remit spontaneously and require no further treatment ^[7] [14]^ but more commonly people will develop mood disorders, anxiety and substance use disorders ^[2, 7]^. There is strong evidence from a large, prospectively obtained, help-seeking cohort that almost three quarters of CHR have another clinical diagnosis of mental illness that warrants treatment ^[4].^ This is consistent with a recent systematic review that found up to 80% of CHR patients had another diagnosable mental illness, and almost half experienced poor psychosocial functioning up to six years after first seeking help ^[15]^. For these reasons, developing and testing multi-component interventions that address complex intersecting needs in developmentally informed ways, including youth and family engaged intervention development and evaluation projects, reflecting the dynamic nature of youth development are needed for young people with CHR.

The current grant proposal details the design and delivery of a comprehensive, evidence-based, ready-to-go psychosocial and mental health support program, the optimal health program (OHP), to effectively enhance mental health and support functional recovery for individuals with CHR. The OHP is a self-management program, delivered virtually through a web-based portal or in-person, which provides a framework to address the psychosocial and mental health of individuals, adopting a patient-centered approach that empowers the individual to be at the center of their own health management ^[16]^. Developed specifically to support people with mental health issues, while also encompassing physical health and substance use management strategies, the OHP comprises psychosocial supports and development of a skill-base designed to build resiliency and maintain optimal health (please see the ‘design and methods’ section for more information about the intervention) ^[16]^. Our previous and ongoing work demonstrates the program’s success in improving depression, anxiety, and quality of life in community mental health settings, as well as in individuals with chronic physical illnesses (see preliminary data below). Failure to provide adequate and timely mental health support to CHR individuals is a missed opportunity to avert progression to more severe outcomes, at which stage affected individuals are more likely to engage with acute mental health services. Given the high prevalence of poor functioning within the CHR population and the pivotal developmental stages CHR presents, effective treatment is likely to deliver substantial economic and social gains, reducing disability and enhancing productivity. OHP-CHR, co-developed by youth for youth, may improve long-term functional outcomes, including vocational and educational outcomes, and enhance quality of life, while reducing reliance on institutionalized care. We argue that virtual delivery of OHP and the complementary digital toolkit are innovative and critical aspects of the proposal that enable access and asynchronous support, especially considering the constant uncertainty and ongoing restrictions related to the COVID-19 pandemic.

## Study Intervention

The OHP intervention will comprise a psychosocial management program that will be adapted to the CHR population and will be accompanied by a structured workbook, all accessed virtually through a web based digital portal. Sessions are approximately 1 hour in duration and held weekly for 6 weeks, and every two weeks for the remaining 6 weeks. The OHP has three components: 1) assessment and engagement; 2) therapy sessions, and 3) maintenance integration:

1. **Assessment/engagement**. The assessment aims to screen for issues that may act as barriers to treatment and to develop a pathway that maximizes the individual’s engagement in the program.
2. **Therapy sessions**. The cornerstone of the OHP intervention is the one-on-one based work which run over the intervention period, followed by a relapse prevention component (final session). Sessions are based on a newly constructed version of the stress vulnerability model (stress vulnerability/self-efficacy), utilizing individual self-efficacy and self-reliance as part of the process. The OHP intervention provides core components of therapeutic interventions that have established efficacy across a wide range of mental health diagnoses. These consist of providing psychoeducation, coping and relapse prevention strategies, and other skills that can be usefully employed to promote positive wellbeing and ongoing mental health maintenance. The OHP has a specific module focusing on alcohol and other drugs; covering stages of change, impact of substance use, implementation of harm minimization strategy, engaging with support groups, change enhancement and problem-solving.
3. **Maintenance integration**. Via an individualized OHP journal accessible through the web based digital portal, participants maintain a journal that can chart stressors, early warning signs, coping strategies, supports and other factors that influence the course and maintenance of their health. It places them at the center of their treatment and provides them with effective skills to maintain wellbeing, manage psychological distress and the ability to facilitate good communication between themselves and others involved in the maintenance of their mental health (e.g., clinicians, family physicians, mental health workers). The journal and the digital portal/toolkit thus: a) allow the participant to identify the supports and services they can draw upon; b) facilitate on-going skills development and provides empowerment and ownership of health and wellbeing; and c) allow monitoring of mental health and physical health parameters over time.

## Clinical Data to Date

We have demonstrated the feasibility of OHP in both a completed trial in a community mental health setting ^[16]^ and our ongoing trials across major chronic diseases. In addition, we have built a virtual version of OHP through a web-based portal (optimized for smart phones/tablets) at CAMH which we will use in this study, however, will adapt the content for the CHR population.

**OHP in a community mental health setting in Australia** ^[16]^

Participants with a diagnosed mental illness receiving treatment from an adult mental health service (*n*=92) were followed for 12 months from initiation of OHP. OHP delivered in a community mental health setting demonstrated significant improvements in health and social functioning and reduced hospital admissions and emergency contacts, with net cost savings per patient.

**KOHP: Kidney Disease Optimal Health Program** ^[17]^

Participants with stage 4 or 5 chronic kidney disease (n=57) were followed over a 12-month period from initiation of OHP. At 12-month follow up, the patients who completed the OHP intervention demonstrated significantly decreased depression compared to the usual care group. The results of the pilot evaluation provide support for the efficacy of the OHP intervention in significantly improving depressive symptoms in patients with an established chronic physical illness. It also demonstrated how OHP can effectively address mental health and physical health issues simultaneously.

**MINDS: The Mental Health in Diabetes Service** ^[18]^

Participants with Diabetes Mellitus (*n*=72) were followed over a 6-month period from initiation of OHP. At 3 and 6-month follow up participants in the OHP group showed a significant improvement in quality of life and a significant decrease in anxiety at the 3-month follow up.

Our pilot studies on OHP for different health conditions have demonstrated feasibility in terms of recruitment, acceptability, and implementation. Participants were drawn from metropolitan and rural areas, demonstrating that OHP can be effectively delivered to diverse communities. These initial findings suggest that OHP has the potential to address depression and anxiety symptoms, whilst improving quality of life. However, OHP has never been adapted and applied to a CHR population.

## Risks/Benefits

There are no anticipated risks to the participants as the intervention is a ‘light-touch’ psychosocial intervention. Anyone identified with acute psychosis or at acute risk of suicide will be assisted to immediately contact local mental health professionals. If required, the research team will accompany the participant to the local mental health service provider. We will provide written protocols including risk management and safeguarding. All assessments will be completed in accessible, private, and appropriate venues that suit the needs and preferences of participants. The appointments will be scheduled at times convenient to participants, considering education, household commitments and employment commitments. Participants will not be exposed to a risk of physical and mental harm that is greater than that typically encountered in normal life and the recruitment materials will direct participants to relevant supports if participation raises any concerns.

# Study Objectives

Conventional CHR intervention studies have focused on transition to psychosis, psychosis-like symptoms, and associated distress with little emphasis on functional outcomes. However, given high rates of psychiatric co-morbidity and poor psychosocial functioning in this population it is critical to identify interventions that reduce distress and disability in young people with CHR. Stress-vulnerability models of psychiatric disorders have led researchers to investigate the role of resiliency as a protective factor in maintaining mental health and well-being. Individuals with CHR have lower resiliency scores when compared to healthy controls, and reduced resiliency in the CHR population is associated with anxiety, depression, and poorer functioning. Enhanced resiliency in individuals with schizophrenia is associated with psychosis symptom remission; however, resilience-building interventions for CHR individuals are yet to be established. The current proposal aims to adapt an existing comprehensive resilience-building psychosocial and mental health support program, the Optimal Health Program (OHP), to a CHR population and collect feasibility evidence for a trial testing its ability to improve functioning, reduce distress, and increase resiliency in individuals with CHR. The OHP is a self-management program, which provides a framework to address the psychosocial and mental health needs of individuals, adopting a person-centred approach that empowers the individual to be at the centre of their own health management, and will be offered virtually. OHP has been associated with improved health and functioning and reduced health care costs in adult mental health service users. OHP could greatly impact the long-term mental health, well-being and functioning of CHR youth; however, the feasibility and preliminary efficacy of delivering an intervention tailored around the varied needs of the CHR group must be established before a larger-scale appropriately powered study is pursued. In accordance with the Strategy for Patient-Oriented Research (SPOR), and the Medical Research Council (MRC) framework for developing and evaluating complex interventions, young people with lived experience will be engaged in the study, from co-design of the study and intervention adaptations, through to knowledge translation.

## Specific Aim 1

To adapt an existing, effective, validated psychological intervention for use in young people with CHR and offered virtually. Young people with lived experience of psychosis will be engaged to review the OHP materials and work with the team to develop CHR-relevant adaptations, such as examples and vignettes, throughout the intervention package. **H1**: OHP can be clinically adapted and refined in a developmentally appropriate way for virtual use among CHR individuals.

## Specific Aim 2

To evaluate the acceptability of OHP and the feasibility of conducting a clinical trial of OHP in individuals with CHR. **H2:** OHP will be acceptable to CHR individuals as demonstrated by qualitative feedback, attendance rates of OHP sessions and the Client Satisfaction Questionnaire (CSQ). OHP for CHR individuals will be feasible as demonstrated by our ability to recruit our desired sample of 30 participants and retain 80% of those recruited by the end of the 12-week study period. Participants’ ability to complete the clinical assessment schedule will also serve as a feasibility indicator.

## Specific Aim 3

To assess the preliminary efficacy of OHP in enhancing resiliency, reducing depression and anxiety, and improving functioning in individuals with CHR in a single-arm exploratory clinical trial. The design has been informed by the MRC framework of developing complex interventions. **H3:** Individuals with CHR receiving OHP will demonstrate enhanced resiliency, reduced depression and anxiety, and improved functioning as a result of treatment.

# STUDY DESIGN

## Overall Design

This is a mixed-methods study employing qualitative and quantitative methods to adapt the OHP intervention and test the feasibility and acceptability of OHP in individuals with CHR. The design of the study has been informed by the MRC framework for developing and evaluating complex interventions ^[19]^. The MRC framework guides researchers in the design, methodology and conduct of research relating to the development of complex interventions.

## Patient Engagement

We will recruit four individuals with lived experience of psychosis to form a youth advisory group to advise on the study design, help build interview guides and advise on iterative adaptations of OHP. We will present the OHP model, explore attitudes and beliefs about the support needs of youth with lived experience of psychosis, experiences of accessing and engaging with treatments, perceptions of psychosocial interventions, impact of illness and priorities/preferences for delivery including session duration, location, practitioner preference and role of the caregiver to inform developmentally appropriate interventions. The youth advisory group will also help develop consent and demographic forms to ensure a youth friendly approach to the project. We will discuss study findings with the youth advisory group and collaborate with them in knowledge translation through co-authorship of study results.

## Quantitative methods

Single arm, pre/post design intervention study. The OHP intervention will be delivered over 12-weeks. Measures will be completed at study entry and repeated immediately post-treatment at 12-weeks. Assessments are clinician rated and will be completed with a trained research assistant.

## Qualitative methods

The sample size for the qualitative component has been determined by the principle of saturation ^[20]^; we anticipate that 15 post-intervention interviews will need to be conducted ^[21]^. Views of the OHP intervention, treatment target preferences, and experiences of assessment schedules will be obtained in individual, semi-structured interviews at intervention completion. Interview data will also be collected on usability of the virtual platform, experiences participating remotely/technical experience, overall perception of the program, and barriers/facilitators to participation. An interview guide will be developed from existing literature, together with feedback from the youth advisory group, and will consist of key questions with prompts to enquire further. All interview data will be transcribed verbatim. Data will be analysed using the five stages of framework analysis ^[22]^. Rigour will be ensured by maintaining an audit trail to foster dependability of the process. Confirmability and credibility will be assured by a team approach to data analysis and discussion of the emerging themes. Consensus of the final theoretical framework, reflexivity, and the impact of the researchers on recruitment, data collection and analysis will also be considered ^[23]^.

## Outcome Assessments

**Sociodemographic and medical comorbidity assessment:** Demographic and medical comorbidity assessments, and current medication use will be gathered at baseline. Any changes to medication will be recorded at intervention completion.

**Feasibility and acceptability outcomes:** These have been informed by the MRC framework for developing and evaluating complex interventions ^[19]^. Feasibility outcomes will include recruitment, adherence, retention, attrition, and completeness of outcome schedule at each time point. Acceptability will be informed by the Client Satisfaction Questionnaire (CSQ-8) ^[24]^. Semi-structured interviews at the end of the intervention will also provide feasibility and acceptability and data.

**Psychiatric assessment:** Psychiatric diagnoses will be confirmed using the Structured Clinical Interview (SCID) for DSM-5 at baseline. Clinical assessments will be completed at baseline and 12-weeks. We will collect data on resiliency (Connor-Davidson Resilience Scale; CD-RISC) ^[25]^, symptom severity (SIPS; Prevention through Risk Identification, Management ^[26]^, and Education Screen – Revised ^[27]^; Calgary Depression Scale for Schizophrenia^[28^]; State and trait anxiety inventory^[29]^),;, global functioning (Global Functioning: Social and Role Scales ^[33]^; The Self-report World Health Organization - Disability Assessment Schedule (WHO-DAS 2.0) ^[34]^), and cognition (MATRICS Consensus Cognitive Battery; MCCB ^[35]^). Additional outcomes may be identified by the youth advisory group.

# PARTICIPANT SELECTION AND WITHDRAWAL

## Target Population

We will recruit 30 CHR participants, with a focus on diversity across genders, ethnic origins, age, and other sociodemographic characteristics. All participants will be assessed at baseline and 12-weeks. We will recruit youth (age 16 – 29 years). The sample size has been informed by published literature, which suggests 24-50 participants for feasibility trials ^[36]^ ^[37]^.

## Participant Recruitment and Screening

Study participants will be recruited from the Slaight Family Centre for Youth in Transition (SFCYT) via two mechanisms: 1) the Slaight centralized clinical research recruitment strategy in SFCYT where a core team of clinical research staff identify and engage all patients receiving early psychosis services in clinical research, providing streamlined triaging of patients to appropriate studies; 2) the CAMH-wide Clinical Engagement and Research Recruitment (CLEARR) infrastructure that enables early identification and engagement in research for all new patients presenting for care at CAMH.

As part of the CLEARR process, a delegated, CLEARR approved Research Coordinator/Physician will identify potential participants and notify the research team and the participant’s clinician about the participant’s eligibility to participate in the study. The clinician will then ask the participant if they would be willing to meet with a study team member about participating. Only with participants’ agreement will they be approached.

The CLEARR coordinator/physician will access personal health information (PHI) in participants’ health records to determine potentially eligible participants for the study.

In case of referrals from clinics, recruitment for the study will be initiated by the clinical team who is treating the potential study participant. The treating physician/clinical care team will not obtain consent. They may identify potential research participants and obtain verbal permission from these potential participants for a member of the research team to approach them. Potential participants who indicate an interest in hearing more about the study and provide verbal consent to be contacted will be contacted by a member of the research team who will engage them in an informed consent process.

We will also contact former research participants who participated in previous studies or are currently participating in parallel studies, and agreed to hear about future research opportunities and consented to be re-contacted. To avoid cold calling, the participants will be contacted by someone from the research team that the former participant was involved with.

Research personnel will contact participants according to their preferred mode of communication (email, telephone, SMS text), which will be recorded in the prescreening/subject log. In the case of text messaging, personnel will only contact participants in order to schedule or remind participants of upcoming appointments.

All patients will be given the option to participate. Participation in the study is voluntary. The decision to participate will not affect patients’ receipt of treatment or clinical services. Participants will be informed that they have the option of terminating their participation at any time, without consequence and that no new data will be collected on them.

Participants will be recruited through the Slaight Centre for Youth in Transition’s centralized recruitment process. All subjects will be pre-screened with the Slaight REB approved “pre-screen form” that is implemented across all SFCYT studies to allow for alignment and data sharing and recruitment; this data will be entered into a password protected and restricted pre-screen tracking database. We conduct case review meetings twice a week to review recruitment and to identify new patients who may be eligible for research studies. Our centralized recruitment strategy allows us to ‘triage’ and ‘group’ participants into non-competing research studies to encourage referrals among PIs.

Following provision of written informed consent, participants will be assessed for suitability for inclusion in the study based on the inclusion and exclusion criteria. If it is deemed necessary, research staff may review participants’ CAMH medical charts for scheduling purposes and/or to obtain additional information for ongoing eligibility determination and/or obtain other clinically relevant information for research purposes. We will also record information about the participant’s mental health from their charts that we are unable to obtain during their interviews. The investigator or research staff may also request information from the participant’s physician (CAMH/non-CAMH) after obtaining consent for release of personal health information to determine eligibility and obtain clinically relevant information for research purposes.

## Eligibility Criteria

### Inclusion Criteria

The participant must meet all of the inclusion criteria to eligible for this study:

1. Be 16-29 years old
2. Being competent and willing to consent to study participation
3. Meets CHR criteria for a psychosis risk syndrome based on the Structured Interview for Psychosis Risk Syndromes (SIPS) either currently or at some point in the past 3 years.

### Exclusion Criteria

An individual who meets any of the following criteria will be excluded from participation in this study:

1. Diagnostic and Statistical Manual of Mental Disorders (DSM-5) diagnosis of psychotic disorder (e.g., schizophrenia spectrum disorder, mood disorder with psychotic features)
2. diagnosis of intellectual disability previously documented in the patient chart
3. Severe developmental disorder
4. Acute suicidality requiring immediate intervention

## Participant Withdrawal Criteria

Participants are advised in the consent form that they are free to withdraw from the study at any time without prejudice and may be withdrawn at the Investigators discretion.

The study participation will also be terminated should participants develop or are found to have any condition that might compromise safety (e.g. unstable vitals) Other reasons for withdrawing individual participants from the study may include one of the following:

- Major protocol violation
- Participant lost to follow-up
- Withdrawal of consent
- Any participant may be discontinued from the study at the discretion of the investigators if it is deemed to be in the best interest of the participant.

# RESEARCH PROCEDURES

## Research Visits

When possible, assessments will be done virtually. Please see schedule of events below. After informed consent has been obtained; participants will complete screening assessments to determine eligibility; followed by baseline clinical, functional and neuropsychological assessments. The clinical, functional and neuropsychological assessments will be repeated approximately 12-weeks (up to 18 weeks after baseline). These assessments can be scheduled on the same day or separate days depending on participant preference. If participants completed overlapping clinical, cognitive or functional assessments as part of their participation of any CAMH study they may be shared, if the participant agrees (based on participant consent). Additionally, many clinics at CAMH use certain rating scales and collect demographic, educational, and cognitive information as part of routine clinical care. For study participants where this information has already been collected as part of routine clinical care, we will endeavor to utilize this already collected information rather than repeating these assessments with participants, if the participant agrees (based on participant consent). Sharing information and assessments will reduce participant burden both by shortening research assessment duration and reducing the stress that can occur with repeated questions on the same topics. Consent for this will be obtained as part of the informed consent procedures for this study.

**Clinical Assessments**

To reduce participant burden, where possible clinical assessments will not be repeated if administered in another study (as outlined above).

**Categorical Assessments**

The Structured Clinical Interview for DSM-5 (SCID-5) ^[32]^ will be used for all participants. The SCID is a semi-structured diagnostic interview designed to assist clinicians, researchers, and trainees in making reliable DSM-5 psychiatric diagnoses. Can be administered virtually. [Approximately 60-120 minutes to complete]

**Dimensional Assessments**

**Psychosis Symptoms**

1. All participants will be administered the PRIME-Revised ^[27]^. This assessment is harmonized with existing measures already utilized in several studies and clinical services at CAMH to minimize participant/patient burden [5 minutes to complete].
2. CHR participants will be administered the Structured Interview for Prodromal Symptoms (SIPS) ^[26]^: The SIPS is a widely used structured interview for diagnosing a CHR syndrome for psychosis and cases of first episode psychosis. It contains a severity rating scale (the Scale Of Psychosis-risk Symptoms, or SOPS), a well-anchored Global Assessment of Functioning (GAF), the DSM-IV schizotypal personality disorder checklist, a brief assessment of the family history of psychosis, and the Criteria Of Psychosis-risk Syndromes (COPS) and Presence Of Psychosis Scale (POPS) and DSM-5 Attenuated Psychosis Syndrome criterion sets. The SIPS rules in/out the current and lifetime presence of psychosis and when psychosis has never been present diagnoses three CHR syndromes. Can be administered virtually. [120 minutes to complete]

**Mood Symptoms**

1. Calgary Depression Scale for Schizophrenia (CDSS)^[28]^ measures the severity of depression symptoms. It is of a 4-point likert type scale with nine items with higher score indicating higher severity. Analyses resulted in a final set of of 9 diagnostic items; each item is accompanied by a brief description. Items are rated on a 4-point scale ranging from 0 (Absent) to 3 (Severe). Survey administration can be done virtually. [5 minutes to complete]

**Anxiety Symptoms**

1. The State-Trait Anxiety Inventory (STAI)[^29]^ is a commonly used measure of trait and state anxiety. It has 20 items for assessing trait anxiety and 20 for state anxiety. All items are rated on a 4-point scale Higher scores indicate greater anxiety. Survey administration can be done virtually. [5 minutes to complete]

**Functioning**

1. The Self-report World Health Organization - Disability Assessment Schedule (WHO-DAS 2.0, self and proxy versions) - a 12-item generic assessment instrument for health and disability ^[34]^. This tool produces standardized disability levels and profiles directly linked at the level of the concepts to the International Classification of Functioning, Disability and Health (ICF), and covers six domains of functioning: cognition, mobility, self-care, getting along, life activities, and participation. Can be administered virtually. [5 minutes to complete]
2. Global Functioning: Social and Role Scales - brief clinician-administered measures of social and role functioning, respectively, administered as a semi-structured interview with detailed anchors for ratings that address functional difficulties typically experienced by youth with subsyndromal PSS ^[33]^. Can be administered virtually. [10 minutes to complete]

**Cognition**

1. The MATRICS Consensus Cognitive Battery (MCCB) (is intended to provide a relatively brief evaluation of key cognitive domains relevant to schizophrenia and related disorders. The MCCB includes ten tests that assess seven cognitive domains: (1) Speed of Processing; (2) Attention/Vigilance; (3) Working Memory; (4) Verbal Learning; (5) Visual Learning; (6) Reasoning and Problem Solving; and (7) Social Cognition. Requires in person administration for standardization. [60 minutes to complete]

**Resiliency**

1. The Connor-Davidson Resilience Scale (CD-RISC) ^[25]^ This is 25-item self-report rating scale designed to assess resilience, with higher scores being an indicator of high resilience. Each item is rated on a 5-point scale ranging from not true at all or zero to true nearly all of the time or four. The total possible scores range from 0–100. [10 minutes to complete]

## Schedule of Events

| **Procedures** | Screening | Baseline | OHP Intervention (12 weeks) | Follow up Visit |
| --- | --- | --- | --- | --- |
| Informed consent | X |  |  |  |
| Inclusion/Exclusion Determination | X |  |  |  |
| SCID for the DSM-5 | X |  |  |  |
| Connor-Davidson Resilience Scale |  | X |  | X |
| SIPS | X |  |  | X |
| Prevention through Risk Identification, Management and Education Screen – Revised |  | X |  | X |
| Calgary Depression Scale for Schizophrenia |  | X |  | X |
| The State-Trait Anxiety Inventory |  | X |  | X |
| Global Functioning: Social and Role Scales |  | X |  | X |
| WHO-DAS 2.0 |  | X |  | X |
| MATRICS Consensus Cognitive Battery (MCCB) |  | X |  | X |
| Optimal Health Program |  |  | X |  |
| Complete Case Report Forms (CRFs) | X | X | X | X |
| Client Satisfaction Questionnaire |  |  |  | X |
| Adverse Event Log | X | X | X | X |

# STATISTICAL PLAN

## Sample Size Determination

There will be 30 participants enrolled in this 12-week single-arm trial. We aim to recruit 30 participants to account for 20% loss to follow up ^[39]^. The sample size was informed by published literature, which suggests 24-50 participants for feasibility trials ^[36]^ ^[37]^. The proposed sample size will provide reasonably reliable quantitative estimates for the targeted feasibility measures. The margins of error are ±12.7% for recruitment rate, ±14.3% for adherence/retention/attrition, and ±12.0% for completeness of outcomes. For Aim 3, the minimum detectable effect size for a pre-post test is 0.60 (Cohen’s D).

## Statistical Methods

Data will be analyzed principally using descriptive statistics to assess rates of recruitment, retention, intervention fidelity, and treatment response. Distributional characteristics of the outcome measures will be assessed for ceiling and floor effects and rates and patterns of missing values. The primary trial outcome will be impact of OHP on functioning, via the Global Functioning: Social and Role Scales. Our prior work with youth has informed us that functioning is more relevant to them than symptom severity ^[40]^. All participants will be assessed at baseline and study completion. The analysis will run a pre-post test to assess for any significant improvements following intervention; given the small sample size, appropriate to a feasibility trial, we will also calculate effect sizes (Cohen’s *d*), which we will compare with the effect sizes from previous OHP trials to gain a sense of the effect that might be achieved in the full-scale trial.

# SAFETY AND ADVERSE EVENTS

Adverse Events (AEs) and Serious Adverse Events (SAEs) will be documented and reported as mandated by current regulations. AEs will be assessed at each study visit. Any AEs will be followed up to resolution or appropriate resolution will be taken as per the direction of the QI. All AEs, whether reported by the participant or observed by study staff/investigators, will be recorded on the AE log along with a brief description, start date/resolution date and any action taken. The AE log will be initialed by the QI, who will make the determination on the seriousness, relationship and expectedness of the AE to the investigational drug/study procedures. Where the event is deemed to meet CAMH REB reporting criteria, the QI will notify REB in accordance with CAMH REB’s reporting requirements and timelines.

# DATA MANAGEMENT, RETENTION AND INTEGRITY

The basic protection against risk in this study will be provided by Dr. Husain (study PI and QI). He will have primary responsibility for monitoring of participants during the entire time they participate in the study. The PI will meet weekly with study personnel to review accrued data, data confidentiality, and adherence to protocol design, recruitment, and participant complaints. During meetings, the study PI will also review the enrollment data, the accrual and integrity of clinical, and neurocognitive data, and any adverse event associated with the various components of the study. Based on these reports, we will determine if there has been any change in the benefit-to-risk ratio of the clinical and cognitive assessment components of the study.

Participant Study File (PSF) will be completed for each participant enrolled in the study. All information recorded on the PSFs for this study will be considered the participant’s source documentation. Source data from the PSF will be collected and documented on paper and/or using the “Electronic Data Capture” solution (REDCap). A participant screening and enrollment log, noting reasons for screen failure, where applicable, will be maintained for all participants.

Study data will be entered in a secure database (REDCap). At point-of-entry, data values will undergo consistency edits (e.g., ID validation, range verification, duplicate detection) and personnel will be required to correct errors. Data management staff will run logic error programs to check for accuracy and irregularities within and across data structures. Quality assurance checks will be conducted daily by the personnel, as well as biweekly by data management staff. To reduce the incidence of missing data during data acquisition, we will use a number of strategies available in REDCap, including: marking data fields as required and use of the Data Quality module to run regular queries for ALL missing data. This will alert study personnel/PI of missing values and regular data audits using REDCap’s Data Resolution Workflow, allowing a data auditor to open queries (based on the Data Quality Module) for data entry personnel to respond to (and leaving an audit trail that can be tracked). For the relevant assessments, the study REDCap database will also retain form statuses to reflect whether an assessment was collected in-person or virtually.

The hard-copies of data will be stored in a locked filing cabinet in a locked office to further protect participant anonymity. Data auditing, entry, and quality control will be carried out routinely. Regularly scheduled communication between the study team and the PI will clarify any inconsistencies and ambiguities in the data. Additional communication will be conducted as needed.

All data pertaining to a participant’s involvement in this study will be coded and stored securely (in locked offices or secure database/server). This information will only be accessible to the research team. In unusual cases, a participant’s research records may be released in response to a court order. If the research team learns that a participant or someone with whom the participant is involved with is in serious danger or harm, an investigator will inform the appropriate agencies as per legal or regulatory requirements.

During the duration of the study, data and all appropriate documentation will be maintained according to current regulations, and stored for a minimum of 15 years after the completion of the study.

# ETHICAL CONSIDERATIONS

## Research Ethics Board (REB) Approval

Research Ethics Board (REB) approval will be obtained prior to beginning any research-specific procedures. Following initial ethics approval, ongoing ethical approval will be maintained and the clinical trial will undergo REB review at least annually, in accordance with regulatory and REB requirements. The clinical trial will be conducted in accordance with the REB-approved study documents and the determinations (including any limitations) of the REB, and in compliance with REB requirements.

Whenever new information becomes available that may be relevant to participant consent, a consent form and/or consent for addendum will be presented to the REB for review and approval prior to its use. Any revised written information will receive REB approval prior to use.

## Informed Consent Process & Documentation

Potential participants who are capable to consent will be approached and provided with information and pertinent details of the study following the ‘Telephone/Email script to schedule study visits’ document.

The decision to participate will in no way influence or restrict treatment services, and the participant is free to withdraw at any time with no negative consequences. The individual obtaining the informed consent will review any points about which the potential participant is unclear, and the participant will be invited to ask questions. Our research staff are carefully trained in strategies for interacting with people with severe mental illness, including speaking slowly and clearly, stopping to summarize frequently, and providing time for questions. They are all supervised by senior staff members.

Informed consent will be obtained in-person or virtually.

In-person consent:

Participant will be assessed for competency to provide informed consent and will review the consent document with the study staff. The research staff will go through the informed consent form (ICF) and will explain the study in detail and answer any questions the participant has. After reading the consent, but before obtaining a signature, all participants who express willingness to provide consent will be queried about the consent form in order to verify that the participant has demonstrated comprehension of the study and consent document and is competent to provide informed consent. Physical copies of the signed ICFs and Attestation pages will be provided to the participants.

E-consent:

Participants will be provided with a read-only copy of the informed consent form (ICF) via REDCap prior to conducting the consent discussion. The link may be used by participants as many times as they wish (it is not single-use). Upon clicking the link, participants will review the landing page, and continue on to the ICF text. The entire contents of the ICF will be displayed according to the current REB approved consent form, minus the signature/attestation page(s). Participants will schedule a 2-way videoconference/Teleconference via the WebEx feature with a CAMH research personnel on the team to have a consent discussion.

Informed consent may be documented using the REDCap e-Consent Framework. Following the consent discussion, the prospective participant will be sent a link to the e-consent via email or the chat feature in WebEx. The participant will complete the e-consent and be provided with the option to download and/or email themselves the signed ICF. If email is chosen, the email will only be used for this purpose (it is not retained by REDCap).

Following the participant signature, the person conducting the consent discussion will complete the Person Conducting Consent Discussion Attestation Page. PDF copies of the signed ICFs and Attestation pages will be retained in the REDCap File Repository. The research team will provide the participant with a copy of the fully signed ICF via email, in accordance with the participant’s wishes.

# PRIVACY AND CONFIDENTIALITY

The Investigator will preserve the confidentiality of participants taking part in the study as mandated by current regulation. All personal study participant data collected and processed for the purposes of this study will be managed by the investigators and their staff. Adequate precautions to ensure the confidentiality of those data, and in accordance with applicable national and local laws and regulations on personal data protection will be used (in accordance with CAMH policies, PHIPA, Tri-Council Policy Statement (TCPS2) and the International Conference on Harmonization Guideline for Good Clinical Practice (ICH GCP) requirements).

There is a potential risk of breach of confidentiality that is inherent in all research protocols. Breach of confidentiality will be minimized by the staff who will maintain research data (identified only by participant code number not related to name or date of birth) in separate charts and a dedicated password protected electronic database. A list of participant names, their ID numbers, and information about how they can be reached will be kept in a separate locked cabinet with access only to study personnel authorized by the PI. Procedures have been established, and will be followed, to minimize the risk of breach of confidentiality. Procedures to maintain confidentially include: (1) formal training sessions for all research personnel emphasizing the importance of confidentiality; (2) specific procedures developed to protect participants’ confidentiality, and (3) formal mechanisms limiting access to information that can link data to individual participants. All information obtained from participants will be kept as confidential as possible. Computer based files/data will be entered into password-secured databases and paper-based files will be stored in a secure location. These data will only be accessible to personnel involved in the study and they will abide by confidentiality regulations of the REB. The ethics committee granting approval to this study will be granted direct access to the study participants’ original medical records for verification of study procedures and/or data, without violating the confidentiality of the participants, to the extent permitted by the law and regulations.

Research data gathered as part of this study may be shared and provided to other investigators working with the study team for the purpose of data sharing. If participants are enrolled in multiple studies, their research data will be shared across studies to reduce participant burden and avoid duplication of procedures. Only investigators/research team affiliated with the study team will have access to secured files and/or research data and will be well-informed regarding the protection of participants’ rights to confidentiality.

Furthermore, investigators collaborating with the study team will have access to the research data collected during the study for the purposes conducting secondary analyses about mental illnesses, such as ASD, depressive disorders, psychotic disorders, bipolar disorders, anxiety disorders, sleep disorders, etc. These data will be de-identified and not contain any PHI.

Participants will not be identified by name in any publication or presentations at meetings of research results. Results will be published as group data without the use of characteristics that would identify individual participants.

All study-related records will be kept for at least 15 years according to current regulations.

# FUNDING

This study is funded by the Miner’s Lamp Innovation Fund (PI: Dr. Omair Husain)

# REFERENCES

1. Andreou, C. and S. Borgwardt, *Structural and functional imaging markers for susceptibility to psychosis.* Mol Psychiatry, 2020. **25**(11): p. 2773-2785.

2. Lin, A., et al., *Outcomes of nontransitioned cases in a sample at ultra-high risk for psychosis.* Am J Psychiatry, 2015. **172**(3): p. 249-58.

3. Taylor, P.J., P. Hutton, and L. Wood, *Are people at risk of psychosis also at risk of suicide and self-harm? A systematic review and meta-analysis.* Psychol Med, 2015. **45**(5): p. 911-26.

4. Fusar-Poli, P., et al., *Comorbid depressive and anxiety disorders in 509 individuals with an at-risk mental state: impact on psychopathology and transition to psychosis.* Schizophr Bull, 2014. **40**(1): p. 120-31.

5. Alvarez-Jimenez, M., et al., *Road to full recovery: longitudinal relationship between symptomatic remission and psychosocial recovery in first-episode psychosis over 7.5 years.* Psychol Med, 2012. **42**(3): p. 595-606.

6. Fowler, D., et al., *Social recovery therapy in combination with early intervention services for enhancement of social recovery in patients with first-episode psychosis (SUPEREDEN3): a single-blind, randomised controlled trial.* Lancet Psychiatry, 2018. **5**(1): p. 41-50.

7. McGorry, P.D., et al., *Beyond the "at risk mental state" concept: transitioning to transdiagnostic psychiatry.* World Psychiatry, 2018. **17**(2): p. 133-142.

8. WHO, *Preventing suicide: A global imperative.* 2014, Geneva.

9. Kelleher, I., et al., *Psychotic symptoms and population risk for suicide attempt: a prospective cohort study.* JAMA Psychiatry, 2013. **70**(9): p. 940-8.

10. Hutton, P. and P.J. Taylor, *Cognitive behavioural therapy for psychosis prevention: a systematic review and meta-analysis.* Psychol Med, 2014. **44**(3): p. 449-68.

11. Devoe, D.J., et al., *Interventions and social functioning in youth at risk of psychosis: A systematic review and meta-analysis.* Early Interv Psychiatry, 2019. **13**(2): p. 169-180.

12. van der Gaag, M., et al., *Preventing a first episode of psychosis: meta-analysis of randomized controlled prevention trials of 12 month and longer-term follow-ups.* Schizophr Res, 2013. **149**(1-3): p. 56-62.

13. Stafford, M.R., et al., *Early interventions to prevent psychosis: systematic review and meta-analysis.* BMJ, 2013. **346**: p. f185.

14. Polari, A., et al., *Clinical trajectories in the ultra-high risk for psychosis population.* Schizophr Res, 2018. **197**: p. 550-556.

15. Beck, K., et al., *Clinical and functional long-term outcome of patients at clinical high risk (CHR) for psychosis without transition to psychosis: A systematic review.* Schizophr Res, 2019. **210**: p. 39-47.

16. Gilbert, M.M., et al., *Controlled clinical trial of a self-management program for people with mental illness in an adult mental health service - the Optimal Health Program (OHP).* Aust Health Rev, 2012. **36**(1): p. 1-7.

17. Knowles, S.R., et al., *Design and protocol for the Dialysis Optimal Health Program (DOHP) randomised controlled trial.* Trials, 2016. **17**(1): p. 447.

18. O'Brien, C.L., et al., *The Mental Health in Diabetes Service (MINDS) to enhance psychosocial health: study protocol for a randomized controlled trial.* Trials, 2016. **17**(1): p. 444.

19. Skivington, K., et al., *A new framework for developing and evaluating complex interventions: update of Medical Research Council guidance.* BMJ, 2021. **374**: p. n2061.

20. Saunders, B., et al., *Saturation in qualitative research: exploring its conceptualization and operationalization.* Qual Quant, 2018. **52**(4): p. 1893-1907.

21. Sebele-Mpofu, F.Y., *Saturation controversy in qualitative research: Complexities and underlying assumptions. A literature review.* Cogent Social Sciences, 2020.

22. Spencer, L., Ritchie, J., Ormston, R., O’Connor, W., and Barnard, M. , *Qualitative research practice : a guide for social science students and researchers*. 2nd ed. 2014, London: Sage Publications Ltd.

23. Tobin, G.A. and C.M. Begley, *Methodological rigour within a qualitative framework.* J Adv Nurs, 2004. **48**(4): p. 388-96.

24. Attkisson, C.C. and R. Zwick, *The client satisfaction questionnaire. Psychometric properties and correlations with service utilization and psychotherapy outcome.* Eval Program Plann, 1982. **5**(3): p. 233-7.

25. Connor, K.M. and J.R. Davidson, *Development of a new resilience scale: the Connor-Davidson Resilience Scale (CD-RISC).* Depress Anxiety, 2003. **18**(2): p. 76-82.

26. Miller, T.J., et al., *Prodromal assessment with the structured interview for prodromal syndromes and the scale of prodromal symptoms: predictive validity, interrater reliability, and training to reliability.* Schizophr Bull, 2003. **29**(4): p. 703-15.

27. Miller, T.J., et al., *The PRIME North America randomized double-blind clinical trial of olanzapine versus placebo in patients at risk of being prodromally symptomatic for psychosis. II. Baseline characteristics of the "prodromal" sample.* Schizophr Res, 2003. **61**(1): p. 19-30.

28. Addington, D., J. Addington, and E. Maticka-Tyndale, *Assessing depression in schizophrenia: the Calgary Depression Scale.* Br J Psychiatry Suppl, 1993(22): p. 39-44.

29. Tluczek, A., J.B. Henriques, and R.L. Brown, *Support for the reliability and validity of a six-item state anxiety scale derived from the State-Trait Anxiety Inventory.* J Nurs Meas, 2009. **17**(1): p. 19-28.

30. Andreasen, N.C., *The Scale for the Assessment of Negative Symptoms (SANS): conceptual and theoretical foundations.* Br J Psychiatry Suppl, 1989(7): p. 49-58.

31. Löwe, B., et al., *Measuring depression outcome with a brief self-report instrument: sensitivity to change of the Patient Health Questionnaire (PHQ-9).* Journal of affective disorders, 2004. **81**(1): p. 61-66.

32. First, M.B., et al., *Structured clinical interview for DSM-IV-TR axis I disorders, research version, patient edition*. 2002, SCID-I/P New York, NY, USA:.

33. Cornblatt, B.A., et al., *Preliminary findings for two new measures of social and role functioning in the prodromal phase of schizophrenia.* Schizophr Bull, 2007. **33**(3): p. 688-702.

34. Kimber, M., J. Rehm, and M.A. Ferro, *Measurement Invariance of the WHODAS 2.0 in a Population-Based Sample of Youth.* PLoS One, 2015. **10**(11): p. e0142385.

35. Nuechterlein, K.H., et al., *The MATRICS Consensus Cognitive Battery, part 1: test selection, reliability, and validity.* Am J Psychiatry, 2008. **165**(2): p. 203-13.

36. Sim, J. and M. Lewis, *The size of a pilot study for a clinical trial should be calculated in relation to considerations of precision and efficiency.* J Clin Epidemiol, 2012. **65**(3): p. 301-8.

37. Julious, S.A., *Sample size of 12 per group rule of thumb for a pilot study.* Pharmaceut Sta, 2005. **4**: p. 287–291.

38. Spitzer, R.L., et al., *A brief measure for assessing generalized anxiety disorder: the GAD-7.* Arch Intern Med, 2006. **166**(10): p. 1092-7.

39. van Ballegooijen, W., et al., *Adherence to Internet-based and face-to-face cognitive behavioural therapy for depression: a meta-analysis.* PLoS One, 2014. **9**(7): p. e100674.

40. Henderson, J., et al., *Youth and family members make meaningful contributions to a randomized-controlled trial: YouthCan IMPACT.* Early Interv Psychiatry, 2021.
