# Supplementary material for: A mixed-methods study to evaluate the feasibility and preliminary efficacy of delivering the optimal health program (OHP) for youth at clinical high risk (CHR) for psychosis: A study protocol
Source: PLoS One. 2024 Jul 18;19(7):e0306968. doi: 10.1371/journal.pone.0306968 (PMC11257342; doi:10.1371/journal.pone.0306968)
Supplement: S1 File — (DOCX) [file pone.0306968.s003.docx]

Consent to Participate in a Research Study

**Study Title**: Optimizing Mental Health for Young People at Clinical High Risk for Psychosis (CHR)

**Lead Researcher**: Dr. Omair Husain MBBS, MRCPsych

Schizophrenia Division

416-535-8501 ext. 36467

**Funder(s):** Miner’s Lamp Innovation Fund

INTRODUCTION

This consent form describes a research study and what it means to take part in it.

It is your choice whether you decide to take part in this study or not. If you do decide to participate, you can change your mind later. No matter what you decide, it will not affect the care or any other services that you receive at CAMH.

Please take as much time as you need to decide. If you’d like to, you can talk about this study with other people (for example, your family, friends, your usual doctor, and other health professionals). Please ask the research team any questions you have.

WHY IS THIS STUDY BEING DONE?

You are being asked if you’d like to take part in this study because you have are considered to be clinically high risk (CHR) for developing psychosis. People who are considered CHR may benefit from early and appropriate interventions to help reduce the risk of developing psychosis.

The purpose of this study is to investigate whether a comprehensive psychosocial and mental health support program, called the Optimal Health Program (OHP), may improve functioning, reduce distress, and build resiliency in people who are considered CHR. OHP has been associated with improved health and functioning in mental health service users.

HOW MANY PEOPLE WILL TAKE PART?

About 30 people will take part in this study at CAMH.

WHAT WILL HAPPEN DURING THIS STUDY?

**Before you begin the study:**

Research studies have strict requirements about who can join to make it safer for participants and the study results reliable. You will complete some tests to make sure that you meet the specific requirements of this study. You may find out that you are not eligible to take part.

**Screening (45 minutes):** During this visit you will: meet the research staff who will explain what is involved in the study; assess eligibility, and answer any questions you may have regarding the study. The research staff will ask you to sign this informed consent form if you decide to participate. A copy of your consent will be either emailed to you securely after your virtual visit, or provided in paper to you at the end of your first in-person visit.

**Initiation of study visit:** If you take part in the study, you will have a study visit that will happen before the 12-week OHP intervention. After visit 1, OHP will be delivered weekly for 6-weeks and every 2-weeks for the next 6-weeks. At each study visit the following will be done:

Each study visit will take about 4-hour. As part of the study visits you will be asked questions about:

- Demographics (ex: age, gender, address)
- Medical history
- Past and/or present mental health symptoms
- You will complete some questionnaires, clinical scales and interviews that will ask about how you are feeling.
- You will also complete some cognitive testing to measure your memory, attention, and other aspects of your cognitive abilities.
- We would also like to ask you about certain events that may have occurred in your life that could be related to the diagnosis of psychosis or experiences that occur in people who are at risk of developing psychosis
- We will confirm contact information for you and others in your life who know how to get in touch with you so that we can reach you throughout the study

**End of study visit:** When you have completed the 12-weeks, you will be asked to come in for a final study visit. This will happen even if you stop the study intervention early (for example, if you decide you no longer want to take part). This visit will be scheduled as soon as possible once you stop the study intervention. The end of study visit will take approximately 4 hours. During the visit, the following will be done:

- You will complete some questionnaires, clinical scales and interviews that will ask about how you are feeling.
- You will also complete some cognitive testing to measure your memory, attention, and other aspects of your cognitive abilities.
- We would also like to ask you about certain events that may have occurred in your life that could be related to the diagnosis of psychosis or experiences that occur in people who are at risk of developing psychosis
- You might be asked to participate in an interview about your experience with OHP. This interview will be semi-structured and qualitative, which means that the interview will have a list of topics to ask you related to your experiences with OHP intervention. The interview will be conducted at CAMH or through Webex and will be audio recorded and transcribed so that we can accurately capture what you say. If you would like to participate, you will be providing your consent for your interview to be audio recorded

WHAT ARE MY RESPONSIBILITIES AS A STUDY PARTICIPANT?

You will be required to:

- Complete all study visits on time
- Plan the dates and times of your visits in advance
- Inform the study coordinator of all events you think might affect your participation in the study
- Inform the study team if anything about your health, prescription or non-prescription medications (e.g. vitamins and herbal supplements) has changed.
- Complete questionnaires when asked to do so

HOW LONG WILL I BE IN THE STUDY?

The OHP program will take place over a duration of approximately 12-weeks. Each session is approximately 1-hour in duration. For the first 6-weeks, sessions will be held weekly and for the remaining 6-weeks, sessions will be held every 2-weeks.

This study should take about 1.5 years to complete participant recruitment and the results should be known in about 2 years.

WHAT OTHER CHOICES MIGHT I HAVE?

You do not have to take part in this study to receive treatment or care at CAMH. If you decide not to take part in the study you can still receive the usual treatment or care.

Please talk to your usual doctor or the research team about the benefits and risks of the other options and whether they may be suitable for you before you decide to take part in this study.

WHAT ARE THE RISKS, HARMS OR DISCOMFORTS?

**Assessments (Memory and Attention Assessments, Clinical Scales, and Interviews):**

Assessments can involve emotional discomfort and possibly fatigue. Some of the assessments, in particular the questionnaires related to mood and emotions may be upsetting for some individuals. If any particular question makes you feel uncomfortable, you may discuss its relevance to the study with the specially trained interviewer. You can also request to take breaks and continue at another time. Participants may choose to skip responses of some questions or choose to reschedule assessments for a later date or time.

**Teleconferencing/Videoconferencing:**

Like other online activities, teleconferencing/videoconferencing technology has some privacy and security risks. While using teleconferencing/videoconferencing, it is possible that information could be intercepted by unauthorized personals (hacked) or otherwise shared by accident. This risk cannot be eliminated. However, CAMH has approved the use of WebEx (a two-way videoconferencing application) for videoconferencing sessions because the appointments take place over a secure encrypted network. We want to make sure that you are aware of this.

WHAT ARE THE BENEFITS?

The researchers do not know whether or not you will benefit from taking part in the study. You may benefit from the close monitoring if your clinical condition. The trial also has the potential to benefit the mental health of the individuals who are participating in the study as hypothesized by the aims of this proposed study. Researchers hope the information learned from this study will benefit other people who are at-risk for psychosis in the future.

CAN I LEAVE THE STUDY?

You can change your mind at any time and decide to not take part anymore (called withdrawal). The research team may ask why you are withdrawing for reporting purposes, but you do not need to give a reason if you do not want to. If you decide to stop, you may also be asked questions about your experience with the study intervention. If you decide to leave the study, please contact the research team to let them know.

If you withdraw from the study, information that was recorded before you withdrew will be kept by the researchers (it will not be destroyed), but no more information about you will be collected without your permission.

CAN MY PARTICIPATION END EARLY?

The researchers may take you out of the study early if:

- Staying in the study might be harmful to you (for example, if you experiences side effects)
- The researchers do not think it is in your best interest to continue
- You are unable to complete all required study procedures
- The study is stopped early or cancelled

Privacy and Confidentiality

**What personal information or personal health information will be collected?**

If you decide to participate in this study, the research team will collect personal information or personal health information for the purposes of the study. Information will be collected directly from you (for example, during interviews and on questionnaires) and from your medical records.

Personal information is information of a personal nature about you that could identify you. Personal health information is information about your physical or mental health or the health care that you receive that could identify you. Information collected for this study may identify you when used alone (e.g. your name) or when combined with other available information about you.

The personal information or personal health information that may be collected, used, and stored in connection with this study will include name, phone number, email address, medical record number, and partial date of birth*,* information about your health including the dates and results of medical tests or procedures, the results of study-specific tests or procedures, sex and/or gender, and race/ethnicity.

Studies involving human participants sometimes collect information on aspects of identity such as gender, and ancestry as well as other characteristics of individuals like income/education/household size because these characteristics may influence how people respond to different interventions, may be useful in ensuring a diverse and representative sample, and provide a more complete picture of who is involved in the study. In this study, these questions are being asked to determine if these factors influence how if and how individuals respond to the OHP intervention.

**How will my personal information be kept confidential?**

Directly identifying information (like your name) will be removed from the rest of the information the research team collects about you and replaced with a code (‘coded data’). The research team will have a list that links your name to your code so that your coded data can be linked back to you if necessary. This list will be kept separate from the coded data in a secure place. Even though the likelihood that someone may identify you from the coded data is very small, the risk can never be completely eliminated. All paper copies, audio recordings, and transcripts will be securely stored in locked offices and on a secure hospital network. All material will be confidential and verbatim quotes that may identify the speaker will not be used in any of the reports from this project.

If the results of this study are published, your identity will remain confidential. It is expected that the information collected during this study will be used in analyses and published/ presented to the scientific community at meetings and in journals. This information may also be used as part of a submission to regulatory authorities around the world to support the approval of the study intervention.

There are some special circumstances in which the researchers may need to share information that you provide and in which your confidentiality may not be protected. Examples of these special circumstances would be if members of the research team have reasonable grounds to believe that disclosing information is necessary to eliminate or reduce a significant risk of bodily harm to yourself or others, if there is reasonable suspicion that a child is at risk of harm or neglect or is witnessing parental violence, if a healthcare professional has engaged in sexual behaviour with a patient or if our files are subpoenaed by a court of law. Should this happen, your information may be shared with the police, healthcare, or protective services.

**What information will be added to my medical records?**

Your participation in this study will also be recorded in your medical record at CAMH. This is for clinical safety purposes. If you participate in this study, information about you from this research project may be stored in your hospital file and in the hospital computer system. CAMH shares patient information stored in our electronic health record with other hospitals and healthcare providers in Ontario so they can access the information if it is needed for your clinical care. The study team can tell you what information about you will be stored electronically and may be shared outside of CAMH. If you have concerns about this, or have any questions, please contact the Information and Privacy Office at 416-535-8501 x33314 or by email at [privacy@camh.ca](mailto:privacy@camh.ca).

**Who may have access to my study records?**

Members of the research team and authorized representatives of CAMH, including the CAMH Research Ethics Board and Research Quality Assurance Office, will have access to your study records (including personal information and personal health information) for use in connection with this study.

For your safety, authorized personnel from CAMH will confirm the name and date of birth that you provide with your government issued identification.

**Will my information be shared?**

Research information gathered as part of this study may be shared with other studies that you are participating in at the Centre for Addiction and Mental Health (CAMH) now or in the future. If you are already enrolled in another study at CAMH, we may use research information from the other study in this study too. This will decrease the amount of time you spend completing questionnaires and other study procedures. We do not think you will experience any negative effects or risks from this. If you would like to know more about this, please feel free to ask the research team.

What else should I know about privacy and confidentiality?

Data collected using the Optimal Health Program web portal resides on MEMOTEXT Corporation servers. CAMH can’t make promises about confidentiality, that the data will only be used for the purpose of this study, or that the data sent by the internet will not be intercepted by unauthorized people (hacked). To mitigate this risk, any data collected through the Optimal Health Program web portal will be encrypted both in transit and at rest.

The security of information sent by e-mail/text cannot be guaranteed. Please do not communicate personal sensitive information by e-mail/text. Let the research team know if you do not want to be contacted by e-mail/text. Email/Text is not routinely monitored outside of work hours. Please do not use e-mail/text to communicate emergency or urgent health matters – please contact your clinician or family doctor. If it is a medical emergency, call 911. By signing this form, you agree that the research team may contact you by email/text for the purposes of this study.

Can my study data be used for other research?

The researchers doing this study may use your data in the future for other research projects. They may share your data with other researchers at CAMH or with collaborators around the world*.* Coded data collected about you from this study may be combined with data collected from other people on other studies, or it may be saved in a database. The research team doesn’t know what this research may be yet, but we think it will be related to future research on individuals at clinical high risk for psychosis*.* You will not be asked or told about these other studies and it is possible that, if you were asked, you would have decided not to take part in these studies.

The results of these studies will not be shared with you. You will not directly benefit from these future studies, but it is hoped that the research may help other people in the future.

Any personal information that could identify you will be removed or changed before the data *or sample* is shared with other people. Data will be kept for 10 years. If you withdraw your consent for future research it may not be possible to delete data that have already been shared.

There is a risk that someone could trace the information back to you. The chance that someone could do this is very small, but the risk may grow in future if people come up with new ways of tracing information back to people.

WILL MY FAMILY DOCTORS/HEALTH CARE PROVIDERS KNOW I AM PARTICIPATING IN THIS STUDY?

Your family doctor will not be told by the research team that you are taking part in the study, but you can tell them if you’d like to.

WILL information BE available online?

A description of this clinical trial will be available on https://clinicaltrials.gov/ct2/home. This website will not include information that can identify you. You can search this website at any time.

IS THERE A COST TO ME?

There may be costs associated with study visits. For example, parking or transportation, child care, or snacks/meals during your stay. The research team will do their best to minimize these extra costs by arranging your research visits around your clinical appointments where possible. We will also provide compensation for the initial study visit and the final study visit.

Will I be able to continue with the intervention after the study is over?

You may not be able to continue with the study intervention after your participation in the study has ended. This may be because:

- The intervention is only available to research participants
- Your health care providers may not feel it is the best option for you

The research team will talk to you about your options.

WILL I BE COMPENSATED?

You will receive $30.00 compensation for the initial study visit and the final study visit.

WHAT ARE MY RIGHTS AS A RESEARCH PARTICIPANT?

If the researchers learn about new information that may be important to your decision to stay in the study they will tell you about it in a timely manner.

You can find out the results of this study once the entire study is complete. Please contact the research team to learn the results.

By signing this form you do not give up any of your legal rights.

You will be given a copy of this signed and dated consent form.

WHAT IF RESEARCHERS DISCOVER SOMETHING ABOUT ME?

We do not expect that the researchers will learn something about you that they didn’t expect. There are no diagnostic investigations that are being completed as a part of this study.

IS THERE A CONFLICT OF INTEREST?

Dr. David Castle is a founder of the Optimal Health Program (OHP), and holds 50% of the IP for OHP; however, he has never gained financially from OHP and any future use of OHP will be under free license to CAMH, as per usual such arrangements

WHO DO I CONTACT FOR QUESTIONS?

If you have questions about this study, or if you experience a research-related injury, you can talk to the researcher who is in charge of the study at CAMH. That person is:

Dr. Omair Husain 416-535-8501 ext. 36467

Name Telephone

If you have questions about your rights as a participant or about ethical aspects of this study, you can talk to someone who is not involved in the study at all. That person is the Chair of the Research Ethics Board (REB). The REB is a group of people responsible for the ethical oversight of this study. The Chair of the REB can be reached by telephone at 416-535-8501 ext. 34020.

**Study Title**: Optimizing Mental Health for Young People at Clinical High Risk for Psychosis (CHR)

SIGNATURES

- All of my questions have been answered,
- I have read each page and I understand the information within this informed consent form,
- I allow access to my personal health information, medical record and research data as explained in this consent form,
- I do not give up any of my legal rights by signing this consent form,
- I agree to take part in this study.

____________________________ ______________________ _________________

Signature of Participant/ PRINTED NAME Date

____________________________ ______________________ _________________

Signature of Person Conducting PRINTED NAME Date

the Consent Discussion

| **Complete the following section only if the participant is unable to read or requires an oral translation**  If the participant is assisted during the consent process, please check the relevant box and complete the signature space below |
| --- |

The person signing below acted as an interpreter, and attests that the study as set out in the consent form was accurately sight translated and/or interpreted, and that interpretation was provided on questions, responses and additional discussion arising from this process.

____________________________ ______________ _________________

PRINT NAME Signature Date

of Interpreter

____________________________ ____________________________

Relationship to Participant Language

The consent form was read to the participant. The person signing below attests that the study as set out in this form was accurately explained to the participant, and any questions have been answered.

____________________________ _________________ _________________

PRINT NAME Signature Date

of witness

____________________________

Relationship to Participant
